# Identification of the Segregation Kinetics of Ultrathin GaAsSb/GaAs Films Using AlAs Markers

**DOI:** 10.3390/nano13050798

**Published:** 2023-02-22

**Authors:** David Gonzalez, Sara Flores, Verónica Braza, Daniel F. Reyes, Alejandro Gallego Carro, Lazar Stanojević, Malte Schwarz, Jose María Ulloa, Teresa Ben

**Affiliations:** 1 University Research Institute on Electron Microscopy & Materials (IMEYMAT), Universidad de Cádiz, 11510 Cádiz, Spain; 2 Institute for Systems Based on Optoelectronics and Microtechnology (ISOM), Universidad Politécnica de Madrid, 28040 Madrid, Spain

**Keywords:** segregation, III-Sb alloys, STEM analyses

## Abstract

For optoelectronic devices from the near to the far infrared, the advantages of using ultrathin III-Sb layers as quantum wells or in superlattices are well known. However, these alloys suffer from severe surface segregation problems, so that the actual profiles are very different from the nominal ones. Here, by inserting AlAs markers within the structure, state-of-the-art transmission electron microscopy techniques were used to precisely monitor the incorporation/segregation of Sb in ultrathin GaAsSb films (from 1 to 20 monolayers (MLs)). Our rigorous analysis allows us to apply the most successful model for describing the segregation of III-Sb alloys (three-layer kinetic model) in an unprecedented way, limiting the number of parameters to be fitted. The simulation results show that the segregation energy is not constant throughout the growth (which is not considered in any segregation model) but has an exponential decay from 0.18 eV to converge asymptotically towards 0.05 eV. This explains why the Sb profiles follow a sigmoidal growth model curve with an initial lag in Sb incorporation of 5 MLs and would be consistent with a progressive change in surface reconstruction as the floating layer is enriched.

## 1. Introduction

III-Sb compounds have unique properties among III–V semiconductors. First, III-Sb materials extend over a wide bandgap range, from 0.1 eV up to 1.8 eV. Second, they offer the possibility of bandgap engineering as they allow switching from type I to type II band alignments [1]. Therefore, they permit the planning of new materials whose effective bandgap can be adapted by design in the whole range from the near to the long infrared, useful for devices such as laser diodes [2], vertical-external-cavity surface-emitting lasers [3], cascade lasers [4], and photodetectors [5] or in solar cells [6].

Different technological targets have demanded manufacturing approaches involving thin layers at the nanoscale level, such as superlattices [7,8,9], quantum wells [10,11], or capping layers of quantum dots [12,13]. However, experimental composition distribution in the layer can largely differ from the nominal one as a consequence of intermixing phenomena, and such variation becomes critical during thin-layer growth [14]. Several growth processes have been identified that may contribute to intermixing in mixed-anion alloys. The first is that Sb is a known segregant; that is, Sb tends to accumulate at the growth front, delaying its incorporation into the film [15,16]. For decades, surface segregation during the growth of IV and III–V semiconductor alloys has been studied both theoretically and experimentally, which has resulted in several models of different complexity and origin. Walter classified the models as atomistic or phenomenological [17]. Phenomenological models aim to fit the experimental profiles to a geometric series [18] or exponential formulae [19,20] by adjusting two or three parameters. Atomistic models are more realistic since they try to simulate individual atomic site exchanges on the surface monolayers between the topmost monolayers during epitaxy [21,22,23]. Comparative analyses point to the fluid three-layer exchange (F3LE) model as a superior option for describing epitaxial segregation in many semiconductor materials [24,25] since it may also take into account the role of atomic steps, kinks, and even surface reconstructions. Such is the case of III-Sb alloys where several findings have suggested that at least the three top layers participate in their surface reconstruction, the structure of which differs from that of the bulk [26].

In general, all models achieve reasonable approximations to the experimental profiles because the simulations have a sufficient number of free parameters to be fitted, but they often fail to describe the extremes of the segregation curves [9,27]. Another point that is often forgotten is the precise definition of the position where the growth of each layer begins. In order to unambiguously identify the Sb segregation process, a marker technique was used in the present study to investigate it during the growth of ultrathin GaAsSb layers since it helps us to clearly identify the existence of different growth regimes as well as the morphological evolution of the profiles as a function of deposition time. The current analysis allows the segregation to be described rigorously using the F3LE model, reducing the number of parameters to be adjusted and demonstrating that the segregation energy varies during the process.

## 2. Materials and Methods

**Figure 1:**
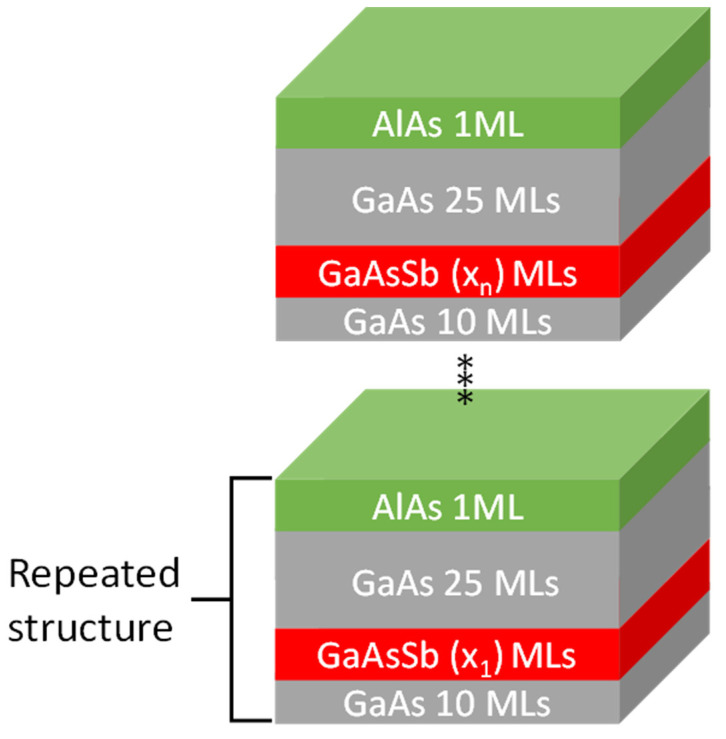
Basic diagram of the sample. Eight periods delimited by AlAs markers contain GaAsSb layers with thicknesses of 1, 2, 3, 5, 7, 10, 15, and 20 MLs enclosed between GaAs spacer layers (10 and 25 MLs).

A sample with 8 periods that included ultrathin GaAsSb layers was grown by solid-source molecular beam epitaxy (MBE) on GaAs(001) n^+^ substrates under As_4_ overpressure conditions. Each period consisted of 10 monolayers (MLs) of GaAs, one layer of GaAsSb, 25 ML of GaAs, plus 1 ML of AlAs used as a marker. The thicknesses of the GaAsSb layers increased successively, with the number of MLs deposited for each period being 1, 2, 3, 5, 7, 10, 15, and 20. Once the periodic structure was completed, a 50 nm GaAs:Be capping layer was deposited. The general structure of the periods is depicted in Figure 1.

Growth conditions were controlled by monitoring the RHEED pattern during growth. The substrate temperature was calibrated by looking at changes in oxide desorption and surface reconstruction. First, as we decomposed native oxide from the GaAs surface at ~580 °C, the RHEED image changed from a diffuse pattern, typical of the rough amorphous oxide layer, to a spotty (2 × 4) diffraction pattern, typical of an ordered As-stabilized (001) GaAs surface. Second, under As_4_ overpressure conditions, the (001) GaAs surface underwent a phase transition at ~510 °C. At this temperature, the RHEED pattern changed from a (2 × 4) to a cubic As-rich (4 × 4) reconstruction. The buffer layers were grown with the substrate temperature of 580 °C and at a measured growth rate of 1 ML/s, the same as the top GaAs:Be layer. Moreover, we used the oscillations of the RHEED pattern during the growth of the buffer to calibrate the growth rate for both GaAs and AlAs. The error in the measurement of the growth rate itself was small, but it can change if the flow is not entirely stable. We can say that it was lower than ±0.1 ML/s. The GaAsSb layers had the same nominal flux for Sb (beam equivalent pressure, BEP_Sb_, of 7·10^−6^ torr), corresponding to a nominal content of 4.5% of Sb. The molecular form of Sb used was Sb_4_ with a standard Knudsen solid-source cell for Sb without cracker. The nominal composition was determined from calibration samples consisting of GaAs/GaAsSb superlattices grown with different Sb fluxes. These samples were grown under the same conditions as the sample under study (same As flux, growth rate, and substrate temperature). With this series, XRD measurements were performed to determine, independently, the thickness of the superlattice layers and the Sb content. In this way, we obtained a calibration for a constant As flux which, for each V-V element ratio (As-Sb), provided a given Sb composition. To separate the periods, after each GaAsSb layer, a 25 ML GaAs spacing layer plus 1 ML of AlAs (used as a marker) was grown. The growth temperature of the whole periodic structure was 480 °C. The design growth rate was 1 ML/s for GaAs(Sb) layers and 0.33 MLs/s for AlAs markers.

The crystal quality of the structure was then analyzed along the direction [110] by high-angle annular dark-field (HAADF) imaging methodology in scanning transmission electron microscopy (STEM) mode, while the Sb composition was quantified by energy-dispersive X-Ray spectroscopy (EDX) using ChemiSTEM technology with four embedded Bruker SDD detectors and processed using Velox^®^ software (version 3.5.0.952). Talos F200X STEM and double-aberration-corrected FEI Titan Cubed^3^ Themis microscopes were used working at 200 kV. In the latter, the sample can be scanned under HRSTEM conditions with a probe size of 0.2 nm and a spatial resolution between 0.07 and 0.09 nm.

## 3. Results and Discussion

### 3.1. Compositional Characterization

**Figure 2:**
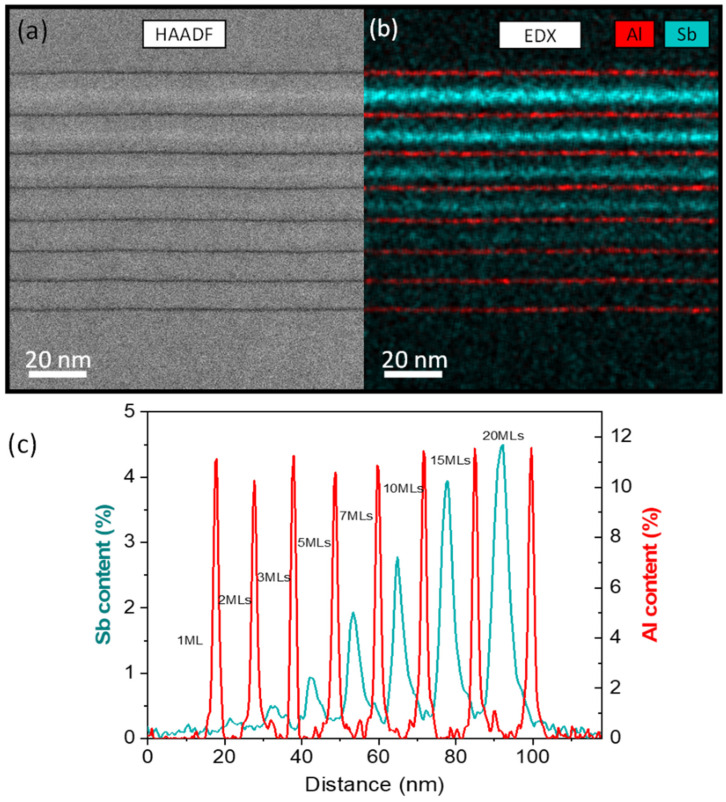
(**a**) Global view of the sample using HAADF imaging conditions. AlAs markers appear as darker stripes while the GaAsSb layers are brighter than GaAs. (**b**) EDX multi-channel mapping showing the Al (red) and Sb (cyan) distribution. (**c**) Average compositional profiles along the growth direction from EDX mappings.

The image on the left of Figure 2 is a low-magnification image of the entire structure using HAADF imaging in STEM mode. The intensity in HAADF conditions roughly depends on the square of the average atomic number of the column, and therefore this method is also known as Z-contrast imaging. Sb and Al atoms have opposite Z-contrast contributions with respect to As and Ga ones, respectively, so the contrast of Sb regions should be distinguishably brighter compared to the GaAs matrix, whereas the Al-rich layers appear darker. In the image, the periods are separated by darker stripes corresponding to AlAs markers, but the brighter contrasts linked to the Sb content are very faint, making it difficult to identify certain GaAsSb layers. All layers grew pseudomorphically with no extensive crystalline defects in the structure. However, although it allowed us to check the structure quickly, this technique, without associated intensity simulations, can only provide qualitative information about the content distribution. Conversely, EDX analyses could provide directly interpretable quantitative mappings for the Al and Sb distributions in the structure. Figure 2b shows a low-magnification multi-channel EDX map of the structure taken at 560 kx, where the blue and red hue intensities correspond to the Al and Sb contents, respectively. The profiles of the average Al (right) and Sb (left) contents from the EDX map along the growth direction are shown at the bottom (Figure 2c). As we can see, not all GaAsSb layers are identified in the profile. Thus, the layer with only 1 ML of GaAsSb is not detected, while the layer with 2 MLs is barely appreciable. On the one hand, the Sb peaks increase with GaAsSb thickness in a quantity that seems to be proportional to the intended Sb deposition. On the other hand, AlAs peaks separate the periods with a maximum content of around 12% Al and thicknesses of around 2 nm, far from the nominal design (100% Al and thickness of 1 ML). This also implies that, even though Al has a lower tendency for atom exchange with Ga [22], the AlAs layers are subjected to considerable intermixing. Thus, AlAs markers widen from the nominal thickness of 1 ML to broader AlGaAs layers, which explains the reduction in the maximum content. Column-resolved EDX elemental maps of AlAs thin films grown under identical conditions, which allow resolving the dumbbell structure [28], have proven that Al is incorporated into the film from the very beginning, so in the following, we consider the appearance of the Al signal in the profiles to be a perfect marker indicating the onset of AlAs layer growth.

**Figure 3:**
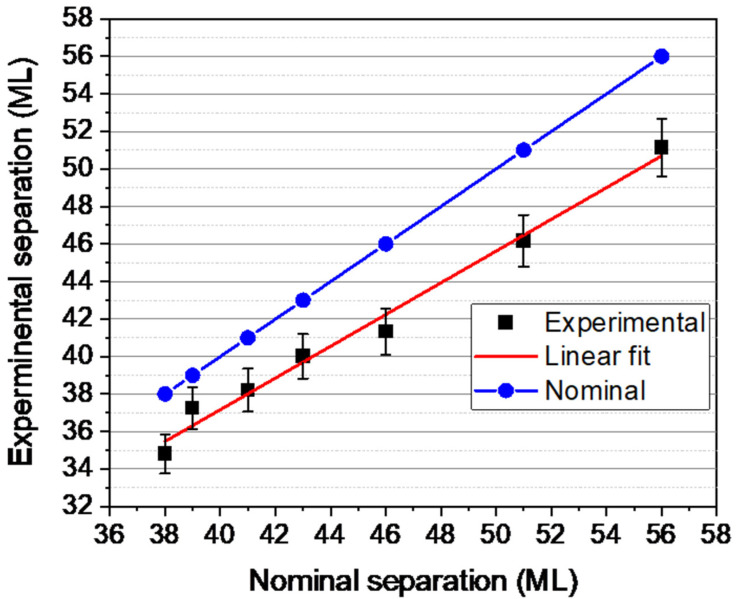
Experimental spacing between AlAs markers (square dots) vs. nominal design spacing. The blue line represents the theoretical nominal spacing with a growth rate (slope) of 1 ML/s. The slope of the red line represents the actual growth rate, which is slightly lower (0.92 ML/s) but remains constant throughout the structure.

In the first step, we used the position of the peaks’ maxima as markers to control the growth rate of the structure, since the Al(Ga)As layer profiles are similar and symmetric in shape. Figure 3 shows the experimental spacings between the AlAs markers, expressed as MLs of GaAs (0.28266 nm), versus the nominal design. Estimation of the tetragonal distortion in the GaAsSb layers showed that the errors in the mean interplanar spacing values are less than 2% even in the thickest layer (20 ML), so these effects were not considered in the spacing measurements. The distances between markers varies for each period since it is a function of the thickness of GaAsSb plus (10 + 25 + 1) MLs corresponding to the bottom and top GaAs spacers plus the AlAs marker. We observe a good linear dependence between the experimental and the nominal distances (growth times), where the square of the correlation coefficient is close to 0.99. This indicates a good control of the growth conditions, especially of the growth rate, during the deposition of the different periods of the sample. However, the distances are always shorter compared to those foreseen in the nominal design (blue line), which are consistent with a slower growth rate, $\dot{G}$, of 0.92 ± 0.03 MLs/s. Overall, we can expect an error of ±1 ML in the position of the markers when comparing periods. Therefore, the period thicknesses are calculated using these measurements which are proportionally shorter (8%) than the nominal ones, mainly affecting the thicker layers. Thus, the thicknesses of the layers with nominal thicknesses of 15 and 20 MLs actually have thicknesses of 13.4 ± 0.4 and 18.2 ± 0.5 MLs, respectively.

**Figure 4:**
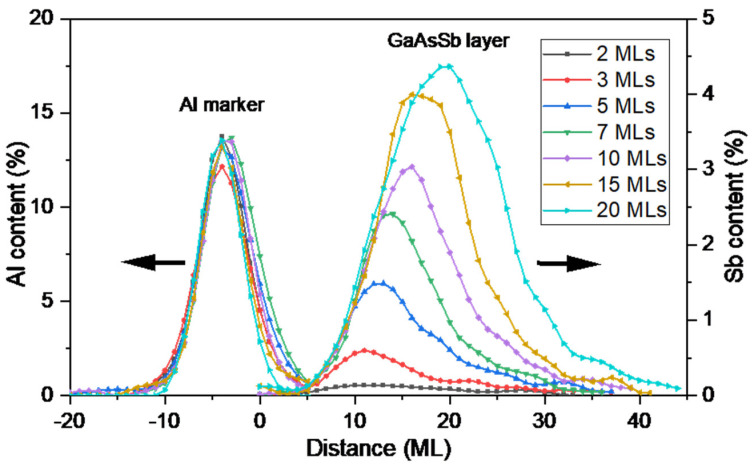
Superposed profiles of Sb and Al content along the growth direction for each period obtained from the high-magnification EDX maps.

To characterize the Sb profiles with higher precision, individual EDX maps for each period were recorded at a magnification of 3.1 Mx. Figure 4 shows the superposition of both the Al and the Sb profiles of all periods, where the right and left coordinates correspond to the Sb and Al contents, respectively. Each profile is the average result of the compositional calculation of the net counts averaged along the growth direction of several EDX maps. Confirming previous results [28], the AlAs marker, supposedly 1 ML thick, becomes an AlGaAs layer, where the signal presence of Al is detected over 10 MLs along the growth direction, arriving near the beginning of the GaAsSb layers. Although the Al peaks have been used to perform the first alignment, a second and finer alignment has been applied to superpose exactly the first ramps of the GaAsSb profiles. Al peaks could be slightly shifted concerning the ideal alignment but within the estimated error of ±1 ML.

A critical point, and the reason for the use of the AlAs marker technique, is to define the onset of the nominal and experimental GaAsSb layer, if different, i.e., if there is a delay in the actual incorporation of Sb into the structure when the Sb flux is turned on. In the plot of Figure 4, we have selected the start of Al incorporation as the position −10 ML before the beginning of GaAsSb growth, which is in the distance 0. Remarkably, the presence of Sb in epitaxy during the first 5 MLs after Sb flux opening is extremely low, below the detection limit of the technique. After this point, the incorporation increases exponentially, except in layers with a thickness of fewer than 5 MLs of GaAsSb, where the increment is lower. The content profiles of the thicker layers follow the same path at the beginning, deviating from it depending on the number of GaAsSb layers deposited.

**Figure 5:**
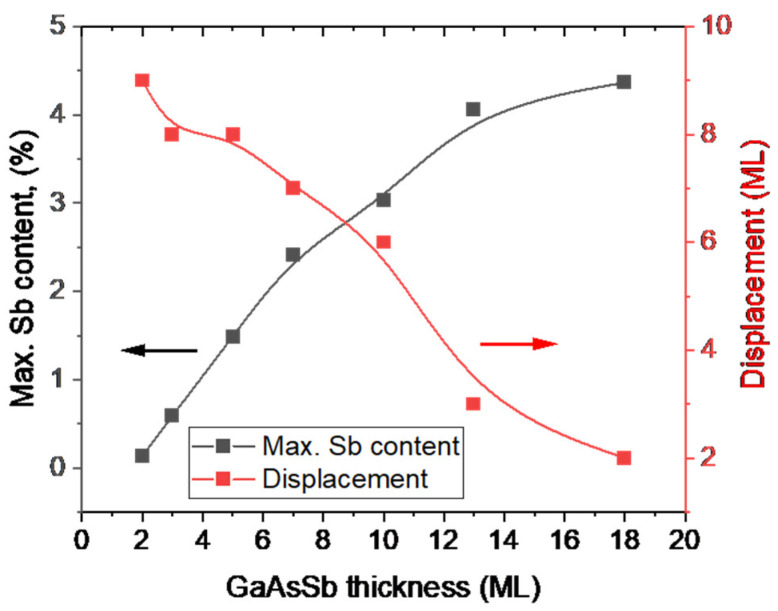
Maximum content (left coordinate, black squares) and displacement (right coordinate, red squares) of the peak maxima with respect to the position when the Sb flux is shut off, i.e., with respect to the nominal end of the GaAsSb layer, for the different periods.

Figure 5 shows for each period the maximum content and displacement of the peak position with respect to the nominal end of the GaAsSb layer. As can be seen, the position and content of the peaks change in each layer. The maximum Sb content in the thickest layer (20 MLs) tends to saturate at about 4.5%. In this case, the peak position is located only 1 ML after the closure of the Sb flux. In the layer with 2 MLs of GaAsSb, the peak position is reached 9 MLs after the closure of the Sb flux (9 MLs of displacement), its content being an order of magnitude lower (0.15%) with respect to the thickest GaAsSb layer.

**Figure 6:**
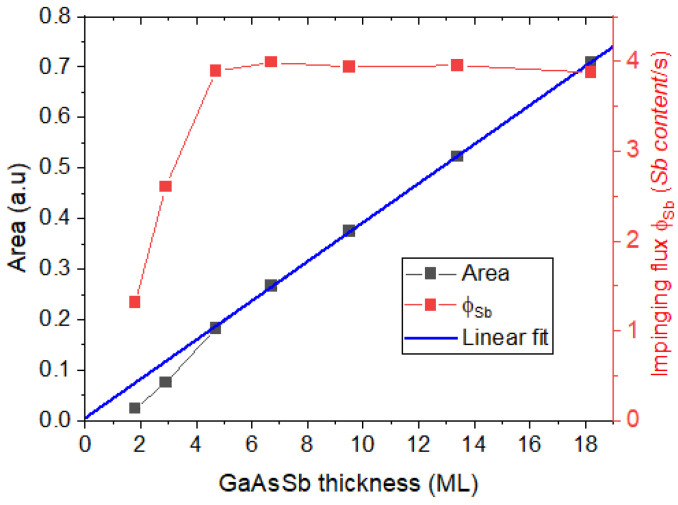
Area under the Sb profiles along the growth direction (left) and the relative impinging Sb flux calculated from every Sb profile (right). Stabilization of growth conditions is only achieved in layers thicker than 5 MLs of GaAsSb.

Although the maximum content is an important parameter, it is not the best for controlling growth quality. Sb is segregated on the growth surface with the formation of a floating layer containing a large percentage of the Sb introduced into the chamber [15,16]. This results in a slow incorporation of Sb into the layer at the beginning, which affects the position and values of the compositional peaks. Still, this Sb-rich floating layer remains after the Sb flux is closed, being nonzero after the deposition of several GaAs monolayers and slowly incorporating into the epitaxy until it disappears. So, the position and value of the composition peak depend not only on the value of the net flux of Sb that arrives at the surface but also on the evolution of this floating layer. In any case, we assume complete incorporation into the epitaxy of the Sb introduced in the chamber at the end of the period growth, so the area under the Sb profile should have a linear relationship with the growth time, i.e., the nominal number of MLs of GaAsSb grown. Figure 6 shows the area under the Sb profiles for all periods, which corresponds to the amount of Sb incorporated in each layer, versus the number of GaAsSb MLs deposited. As can be seen, there is a perfect linear relationship for the case of periods with thicknesses higher than 5 MLs, where the R-squared coefficient is 0.996 and the *y*-intercept is close to zero. This result supports the procedure and assumptions, reinforcing the idea that the structure has a more homogeneous growth than the one at first sight intuited in Figure 3. However, the linear trend fails in the case of GaAsSb layers thinner than 5 MLs, decaying rapidly, and where there is no trace of Sb in the period with a GaAsSb layer of 1 ML. Certainly, the calculated areas are lower in the case of really short Sb flux opening times. We must remember that during the first 5 MLs of GaAsSb growth there is no significant incorporation of Sb into the layer, so we assume that (1) additional Sb desorption may occur during the subsequent growth of GaAs on top of these layers as a consequence of the non-formation of a sufficiently stable floating Sb layer and/or (2) the net impinging flux is lower due to the lack of a simple stabilization of the flow of Sb atoms in the growth chamber in such short times. In any case, it is likely that a small pause in the growth of these extremely thin layers could avoid this effect, but this would imply a change in the growth conditions with respect to the other layers, which would alter the comparison.

Nonetheless, we may calculate the average impinging flux, *ϕ_Sb_*, defined as the net flux of Sb on the surface, that is, the rate of Sb atoms approaching the surface layer diminished by the desorption rate. Therefore, in accordance with the principle of conservation of mass, the net amount of Sb impinging on the surface must be incorporated into the total thickness, i.e., it must be equal to the area of the total profile along the growth direction as

(1) $$\frac{N_{t}}{\dot{G}}\phi_{Sb}=\int_{0}^{z_{f}}x_{Sb}\left(z\right)dz$$

where $x_{Sb}\left(z\right)$ is the fraction of the Sb content in each ML along the growth direction *z* (expressed in ML), *z_f_* is the total length of the experimental GaAsSb layer, *N_t_* is the total number of GaAsSb MLs deposited, and $\dot{G}$ is the growth rate (ML/s). The values of this parameter for each period are plotted in Figure 6. As can be seen, $\phi_{Sb}$ shows a steep growth-type behavior remaining constant from the layer with 5 MLs of GaAsSb onwards. The fact that the mean values of the impingement flux of Sb remain constant for the thicker layers (0.0393 ± 0.0005 *x*/s) suggests that their temporal evolution does not follow a continuous increase. If this were the case, the mean would increase exponentially, and it would take time to reach a steady state. In our experiment, the total amount that arrives at the surface and is incorporated is constant over time, though in the beginning Sb remains only in the floating layer. However, for the case of periods with less than 5 MLs of GaAsSb, the mean impinging flux shows a sharp decrease. The desorbed amount is complete for the 1 ML GaAsSb case but decreases progressively in the 2 and 3 ML periods until the 5 ML case, where a steady state is reached. Furthermore, since the slopes of the first ramps of the Sb profiles are significantly lower in these periods than in the thicker layers, we suggest that this is a kinetic problem due to shorter times to reach stabilization of the floating layer and/or partial pressures in the chamber. In the case of the thicker GaAsSb layers, the system has reached steady-state conditions for Sb impingement, so that all Sb is incorporated into the layer following a linear dependence on the opening time of the Sb shutter. This is consistent with the perfect overlap of all Sb profiles of such layers from the start.

**Figure 7:**
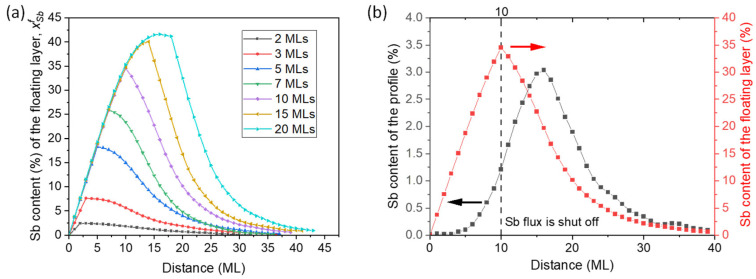
(**a**) Values of the Sb contents of the floating layer during the growth of the different GaAsSb layers. All the layers reach the maximum content at the end of the superficial impinging of Sb, decaying exponentially to vanish completely at the end of the GaAsSb layer. (**b**) Sb content profile of the epitaxy (left, black dots) together with the evolution of the floating layer (right, red dots) for the layer with 10 MLs of GaAsSb.

Indeed, it is possible to calculate the amount of Sb in the floating layer $x_{Sb}^{f}$ in each time (or position where $z=\dot{G}t$). Again, using the conservation of mass principle, the difference between the total amount of Sb that has impinged up to this point (distance, *z*) minus the amount already incorporated into the layer (the area under the profile up to this point) must be the amount in the floating layer, given by

(2) $$x_{Sb}^{f}\left(z\right)=\frac{N\left(z\right)}{\dot{G}}\phi_{Sb}-\int_{0}^{z}x_{Sb}\left(z\right)dz$$

where *N*(*z*) is the number of MLs of GaAsSb deposited up to the distance *z*. Equation (2) converges to Equation (1) at the end of the profile as the amount of Sb in the floating layer approaches zero. Figure 7 shows the evolution of the calculated Sb content of the floating layers for all periods where we can observe regular behavior. For layers of more than 5 MLs, the Sb content of the floating layers increases almost linearly following the same trajectory while the Sb shutter is open, up to contents of around 40% where a stabilization starts to be noticed in the thicker layers. From this point on, a decrease in the Sb content in the floating layer starts to occur, where the slope of this decay ramp increases with the number of MLs deposited. Consequently, the persistence of this floating layer during growth is quite similar in all periods (20–25 MLs after Sb shutter closure) despite the huge differences when comparing the maximum Sb contents. Surprisingly, the compositional values of the floating layer become an order of magnitude higher than the experimentally observed values within the epitaxy.

For a straight comparison of the evolution of the buried and floating layer, Figure 7b plots the values of the time-dependent content of the floating layer for the experimental Sb profile in the epitaxy for the period with a 10 ML GaAsSb layer. In the beginning, only an exceedingly small fraction of the incoming flux is integrated into the film, following a sigmoidal growth model curve, while a high fraction segregates to the floating layer where it rises almost linearly until the Sb deposition flux is shut off. It is important to note that the peak position and content of both curves differ greatly. Indeed, the Sb content at the peak of the floating layer is about 35% and is located at 5 MLs ahead of the deposited Sb peak, with only 3.0%. Both curves converge at the end of the GaAsSb layer, implying the disappearance of the floating layer.

### 3.2. Modeling Segregation

The 3FLE model has been used for describing the segregation in many systems such as SiGe/Si [29,30], GaAsBi/GaAs [31], and GaAsSb/GaAs [14,24,32]. To explain the F3LE model concisely, when the growth of new layer *s* starts, the exchange occurs simultaneously between the top layer *s* and the second top layer *s*-1 and between the second topmost layer *s*-1 and the third topmost layer *s*-2. Once layer *s* is complete, layer *s*-2 is not included in the exchange mechanism anymore and a new layer starts to grow. Therefore, growth and exchange take place simultaneously and an infinite surface diffusion rate is assumed. In each step, a three-differential-equation system is needed to solve for every step [23]:

$$\frac{x_{Sb}^{s}}{dt}=\phi_{Sb}+P_{1}\left(\frac{t}{\tau }-x_{Sb}^{s}\right)x_{Sb}^{s-1}-P_{2}\left(1-x_{Sb}^{s-1}\right)x_{Sb}^{s}$$

$$\frac{x_{Sb}^{s-1}}{dt}=-P_{1}\left(\frac{t}{\tau }-x_{Sb}^{s}\right)x_{Sb}^{s-1}+P_{2}\left(1-x_{Sb}^{s-1}\right)x_{Sb}^{s}+P_{1}\left(1-x_{Sb}^{s-1}\right)x_{Sb}^{s-2}\;-P_{2}\left(1-x_{Sb}^{s-2}\right)x_{Sb}^{s-1}$$

(3) $$\frac{x_{Sb}^{s-2}}{dt}=-P_{1}\left(1-x_{Sb}^{s-1}\right)x_{Sb}^{s-2}+P_{2}\left(1-x_{Sb}^{s-2}\right)x_{Sb}^{s-1}$$

where $x_{Sb}^{i}$ is the time-dependent concentration of the monolayer *i* expressed as a fraction, *τ* is the time to grow one monolayer at a constant growth rate, *ϕ_Sb_* is the impinging flux, and *P*_1_ and *P*_2_ are the exchange probabilities for As-for-Sb and Sb-for-As, respectively. As we demonstrated recently, surface segregation rates are connected for a given temperature, so the two exchange rate probabilities are dependent variables in the system of differential equations [32]. Then, the parameter to fit with the experimental data is the ratio between them, *P*_1_/*P*_2_, which is a function of the difference of the activation energies for each exchange, usually known as the segregation energy,

(4) $$E_{s}=kT\ln P_{1}/P_{2}$$

where *k* is the Boltzmann constant (1.380649 × 10^−23^ J·K^−1^), and *T* is the growth temperature.

In all the fittings, it is necessary to introduce a certain number of parameters such as the time to grow one monolayer, *τ* (the inverse of the growth rate); the number of MLs deposited, *N_t_*; the impinging flux, *ϕ_Sb_*; the exchange probability ratio, $P_{1}/P_{2}$; and the start of growth, that is, *t* = 0, where the Sb flux opening corresponds to the position 0 in the profile. The first two, *τ* and *N_t_*, are supposed to be controlled during the calibration of the growth conditions of the equipment. However, as we have seen above, they can have an error with respect to the nominal design (in our case about 8% by default). In general, segregation energy (i.e., the exchange probability ratio) and impingement flux are the parameters to fit, but a multitude of value pairs can be obtained that fit the experimental profile data if the floating layer content profile is not considered. In general, the composition of the floating layer is an uncontrolled value in most simulations, which are apparently successful, since they focus only on fitting the experimental profiles of Sb into the layer, but in which a huge remnant of Sb remains in the floating layer at the end of the profile, which makes no sense [29]. In our case, as the impinging flux has been measured experimentally, both the experimental profile and the floating layer content ($x_{Sb}^{s-1}+x_{Sb}^{s}$) are considered at every moment during growth, both being zero at the end of the profile.

Besides, a critical issue is the precise choice of the onset of GaAsSb layer growth. In general, almost all models predict a steep linear increase in the composition profile, which decays slowly as the difference between the impinging flux and the surface content decreases. However, although many authors have pointed to the existence of a slow ignition at the beginning of the GaAsSb layer, it is usually neglected in the segregation simulation, and the exact time at which deposition starts is not known [20,32,33]. Our results using the marker technique allow us to know the precise point at which the GaAsSb layer begins (Sb shutter is open), suggesting that the compositional profiles follow a sigmoidal growth curve with a latent period of about 5 MLs before the Sb atoms begin to incorporate exponentially. However, this behavior is difficult to simulate in any kinetic model that assumes constant segregation energy. Indeed, one should consider the possibility that the segregation energy (exchange probability ratio) is different during growth, which is banned in all segregation models. Only Haxha et al. [25] have raised the possibility of explaining the slow increase in profiles at the onset of GaAsSb layer growth that we observe in all periods and which is not described by any of the segregation models. The authors attribute this to changes in the strain energy during the deposition of the first monolayers, which would induce a penalty in the exchange energy. However, this suggestion is difficult to accept because, although the Sb atoms accumulated in this floating layer are bound to the growth surface, it is believed that they do not effectively contribute to the deformation field at the sample surface [34]. In addition, the choice of the initial point is arbitrary, considering only 1 ML in the latent period, and could not explain the behavior from the beginning.

**Figure 8:**
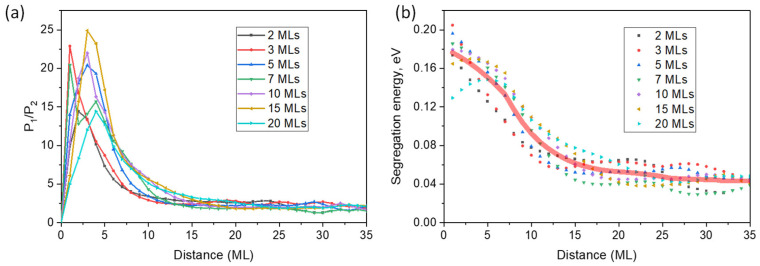
(**a**) The minimum of the exchange rate probabilities ratio and (**b**) the segregation energy during the growth. Steady state is reached in all periods after 15 MLs.

In any case, thanks to the marker technique, we know all the parameters of the model. If we assume that the impinging flux is constant during growth, which we have found to be an exceptionally good approximation at least for layers with more than 5 MLs, one can calculate the segregation energy for each instant during growth, knowing the content of the floating layer at every time. The three coupled differential equations (Equation (3)) were solved numerically layer by layer along the entire Sb profile using the Adams–Bashforth numerical method [35]. In the iterative process, once layer *s* is filled (at time τ), it becomes the new layer *s*-1, and so on. A scan of the probability ratios is made by selecting the minimum value that fits the experimental profiles, assuming a constant impact flux. Figure 8a shows the evolution during the growth of the probability ratio for all periods. As can be seen, all probability ratios in each period evolve from values around 20 at the beginning to converge to a value of 2.17 ± 0.02 from 10–15 MLs deposited, remaining constant until the end of growth. An average segregation energy of 0.05 ± 0.01 eV in the steady-state region is obtained by applying Equation (4) (Figure 8b). The value is slightly lower than the one proposed by Magri et al. [33], 0.07 eV (*P*_1_/*P*_2_ = 2.96), obtained by fitting the Dehease kinetic segregation model [21] to the experimental Sb concentration profiles measured by cross-sectional STM in GaSb/InAs superlattices and which has been used as a standard by numerous authors subsequently [14,24,25,36]. However, as the authors acknowledge, although their fit for the calculation of the segregation energy is excellent, they omit the reconstruction in the first monolayers and do not consider the final value of the floating layer.

Remarkably, the first region of the exchange probability ratio fit shows anomalous behavior. At first, there is a rapid increase up to 5 MLs, followed by a slow decrease to the steady state. In fact, the first ramp, where Sb is not incorporated, *x_Sb_* ≈ 0, is misleading, as it represents the minimum ratio to meet the fit but is consistent with any higher ratio value, such as the one observed at the maximum (around 20). This means that during the first 5 MLs, the segregation energy is remarkably high (higher than 0.18 eV), but slowly decays to a steady state with a segregation energy of 0.05 eV.

This result is compatible with a progressive change in surface reconstruction as Sb accumulation occurs in the floating layer. The As-rich GaAsSb layers show a typical *c*(4 × 4) reconstruction of GaAs(100) surfaces, whereas Sb-rich GaAsSb layers reveal a (1 × 3) reconstruction, detected on GaSb(100) surfaces [37,38]. The latter results in the preferential formation of multiple Sb dimers on the surface due to the higher binding strength of Sb-Sb compared to that of Ga-Sb [39,40]. The higher presence of these Sb dimers favors the substitution of As atoms in the subsurface by Sb, leading to a more efficient incorporation of Sb [41] and, consequently, decreasing segregation energy. As the floating layer becomes more enriched, with Sb contents close to 30%, the percentage of Sb-Sb dimers facilitating Sb incorporation tends to become predominant, governing the segregation kinetics of epitaxial growth.

The advantages of obtaining a greater abruptness of the interfaces as well as the control of the compositional profile during the growth of thin III-Sb layers are evident. According to our results, the growth of ultrathin films presents not only a delay in the onset of the film but also a significant shift in the maximum with respect to the nominal position, and all this seems to be related to the need to obtain from the beginning a sufficiently rich floating film that exceeds a certain critical compositional threshold. This explains why pre-deposition or Sb-soaking stages are usually considered in the growth of this type of layer [42,43]. The idea is to create an interruption in the growth and to expose the pre-growth surface of the layer to an atmosphere rich in Sb. This is expected to promote the formation of a floating layer with an already high Sb content. Although an enhancement of the compositional gradient has already been demonstrated with these processes [32], we do not know to what extent the initial delay in incorporation could be shortened. New research is underway to evaluate and predict the improvements of these strategies using the marker technique.

## 4. Conclusions

The evolution of Sb profiles during the growth of ultrathin GaAsSb films was studied using the AlAs layer marker technique. Firstly, it was observed that there is always a delay of 5 MLs between the opening of the Sb flux and the effective incorporation into the epitaxy in all layers. Secondly, the position and values of the peak contents were found to change as a function of the number of MLs deposited. The rigorous application of the three-layer kinetic model, limiting the number of parameters to be fitted, showed that the segregation energy is not constant throughout the growth (which has never been considered in any model), with an exponential decay from 0.18 eV to converge asymptotically to 0.05 eV at 13–15 MLs from the start.

## Data Availability

Not applicable.
